# Reproductives signature revealed by protein profiling and behavioral bioassays in termite

**DOI:** 10.1038/s41598-023-33252-6

**Published:** 2023-05-01

**Authors:** Fanny Ruhland, Guillaume Gabant, Timothée Toussaint, Matej Nemcic, Martine Cadène, Christophe Lucas

**Affiliations:** 1 Institut de Recherche sur la Biologie de l’Insecte (UMR7261), CNRS – University of Tours, Tours, France; 2 Centre de Biophysique Moléculaire (UPR 4301), CNRS – University of Orléans, Orléans, France

**Keywords:** Behavioural ecology, Chemical ecology, Peptides, Mass spectrometry

## Abstract

Proteins are known to be social interaction signals in many species in the animal kingdom. Common mediators in mammals and aquatic species, they have seldom been identified as such in insects' behaviors. Yet, they could represent an important component to support social signals in social insects, as the numerous physical contacts between individuals would tend to favor the use of contact compounds in their interactions. However, their role in social interactions is largely unexplored: are they rare or simply underestimated? In this preliminary study, we show that, in the termite *Reticulitermes flavipes,* polar extracts from reproductives trigger body-shaking of workers (a vibratory behavior involved in reproductives recognition) while extracts from workers do not. Molecular profiling of these cuticular extracts using MALDI-TOF mass spectrometry reveals higher protein diversity in reproductives than in workers and a sex-specific composition exclusive to reproductives. While the effects observed with extracts are not as strong as with live termites, these results open up the intriguing possibility that social signaling may not be limited to cuticular hydrocarbons or other non-polar, volatile chemicals as classically accepted. Our results suggest that polar compounds, in particular some of the Cuticular Protein Compounds (CPCs) shown here by MALDI to be specific to reproductives, could play a significant role in insect societies. While this study is preliminary and further comprehensive molecular characterization is needed to correlate the body-shaking triggering effects with a given set of polar compounds, this exploratory study opens new perspectives for understanding the role of polar compounds such as proteins in caste discrimination, fertility signaling, or interspecific insect communication.

## Introduction

Communication is one of the keystones of the functioning of societies, which is supported by a diversity of signals^1^. Chemical signals such as pheromones are the most widely used communication system among taxa thanks to the ease of production of a high diversity of compounds^2^. Protein products tend to have high hydrophilicity, which enables dispersion over long distances, making them relevant communication mediators for aquatic species^3^. However, proteins could also have evolved for terrestrial species adaptation, where physical contact with a substrate or between individuals are frequent^3^. Contrary to vertebrates or aquatic species, the implication of proteins in insect social interactions has been fairly neglected by the scientific community so far, despite their potential adaptation for insect societies. In contrast, the role of non-polar compounds in insect has been extensively studied.

Data on protein-based chemical cues for mammals and aquatic species is relatively abundant. In amphibians^4^ and fishes^5,6^, individuals produce protein signals involved in female attraction or sexual behavior. Proteins are also known to be involved in social interactions (mate recognition and attraction, aggressive behavior) in several species of molluscs^7–9^ and crustaceans^10^. In a terrestrial environment, various species, especially rodents and amphibians, use these large molecules as chemical signals during interactions between individuals. In mice, several peptides (i.e., very short proteins) serve as mediators for sexual behaviors. Males can facilitate copulation by directly transferring a peptide (ESP1) to the female vomeronasal organ^11^. On the contrary, sexually immature mice can inhibit the mating behavior of adult males by producing a peptide (ESP22) via their lacrimal glands^12^. Several major urinary proteins (MUP) produced by males are also known to be implicated in individual recognition and aggressive behaviors between males, in the context of territorial markings^13,14^. In amphibians, males produce peptidic pheromones via abdominal, rostral, or mental glands which attract females and enhance their receptivity^15,16^. In terrestrial environments, the lack of volatility and the large molecular weight of proteins limit their dispersion and therefore their perception. So, to increase their efficiency, proteins and peptides are either deposited on a substrate (e.g., at the territorial entrance) or directly transferred by contact to the recipients. This close-contact chemical communication may be promoted in social insects where physical contacts between individuals are frequent within closed structured habitats.

Eusocial insects live in closed environments where individuals constantly interact with the substrate and with their nestmates. They are characterized by a strong division of labor, and especially a reproductive division of labor. Reproductives communicate on their fertility status to maintain this reproductive division of labor^17^. Volatile chemical signals, and also cuticular hydrocarbons compounds (CHCs), are well known to be major components of this reproductive specific signaling, either as a pheromone to induce specific behaviors increasing the colonial inclusive fitness, or as an honest signal announcing the presence of fertile individuals to prevent the development of competitors^18–24^. For decades, the scientific community uncovered a lot of evidence on the CHCs in insect interactions including but not limited to courtship, mating, discrimination, recognition, aggregation, and dominance^25–32^. However, the cuticular waxes of insects also contain proteins which have been identified in several species of cockroaches^33–35^, honeybees^36^, paper wasps^37^, and termites^38^.

Like CHCs, cuticular polar compounds present differences in their compositions according to the sexes or castes which have been revealed in very few species. In the paper wasp *Polistes dominulus*, cuticular polar extracts, which are partly composed of proteins, differ between foundresses and their first emerged daughters^39^. Moreover, the social context seems to influence the protein profiles, *i.e.* orphaned workers have intermediate profiles between queenless and queenright colonies^35^. These specific compositions suggest a role of Cuticular Protein Compounds (CPCs) in social communication such as mate recognition or fertility signaling. Turillazzi et al*.* observed that the cuticular peptides dominulins can be detected and used by foundresses to locate suitable hibernation sites^40^. However, their implication in social interactions still needs to be demonstrated. In this perspective, Bruschini et al*.*^41^ observed that the cuticular polar fraction of *Polistes dominulus* was composed of medium molecular weight compounds ranging from 900 to 3000 Da. They observed that these chemical profiles do not strongly differ between colonies and concluded that these compounds cannot be used alone in nestmate recognition. In the termite *Reticulitermes speratus,* workers recognize eggs by their shape, size, and by a 'Termite Egg Recognition Pheromone' (TERP) composed of an antibacterial protein called lysozyme and a digestive β-glycosidase enzyme^42^. It has been suggested that these proteins could be directly produced by eggs or could be transferred from the reproductives. Variations in CPCs according to castes, sexes, and stages has been identified in three termites species (*Reticulitermes flavipes (santonensis), Kalotermes flavicollis* and *Prorhinotermes simplex*)^38^. CPC profiles from reproductives and non-reproductives differed for the three species but only *P. simplex* showed sex variations. These results clearly suggest a potential implication of CPCs in social interactions and particularly in caste and fertility signaling. We therefore hypothesize that polar compounds such as CPCs represent an alternative to CHCs to support reproductives' signals and we have initiated a first characterization of CPCs along with behavioral experiments.

To explore the potential role of CPCs in reproductive recognition, we have extracted cuticular polar fractions from reproductives and workers. These extracts were used for molecular profiling and tested in behavioral bioassays to measure body-shaking events displayed by the workers. Indeed, it has been recently shown that in the termite *Reticulitermes flavipes*, body-shaking is a vibratory behavior involved in reproductives recognition^18,43,44^. As controls, we also tested live reproductives (no extracts) and polar extracts from workers. The protein profile of the different extracts was determined by mass spectrometry to detect caste- and sex- specific compounds in reproductives and workers. While still quite preliminary in nature, this work combining protein profiling and behavioral assays is the first step in exploring whether polar compounds, and in particular proteins, may contribute to cues or signals in termites, as they do in the animal kingdom.

## Methods

### Study species and laboratory conditions

Stock colonies of *R. flavipes* were collected from 2014 to 2017 in the Oléron island (Charente-Maritime, France) with a distance of at least 300 m between colonies to ensure their independencies^45^. In the laboratory, colonies were maintained in the dark at 26 ± 1 °C with 95 ± 5% of relative humidity. Each colony was kept in a separate plastic box (Star-pack) containing cellulosic ultrapure papers (47 mm diameter; grade 42 Ashless, Whatman)^32^ supplied with wood sawdust. Papers and sawdust were humidified using microfiltered water.

### Behavioral experiments

**Figure 1:**
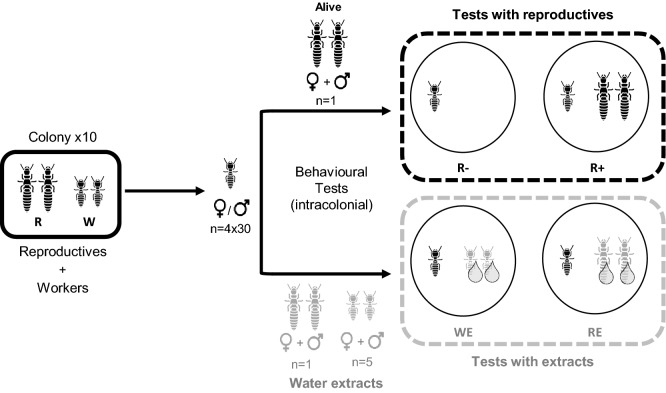
Experimental micro-nests were composed of 30 workers of both sexes in absence of reproductives (R−), in the presence of reproductives (R+), with workers’ extract (WE) or with reproductives’ extract (RE). Each micro-nest has been video recorded twice at 1 h and 24 h after they had been made. Water was used as a solvent and was added to all the treatments. Living individuals and extracts are represented in different grey tones.

For each treatment, thirty workers were isolated from 10 stock colonies containing individuals of every caste. They were sorted on a CO_2_ pad and randomly distributed in 4 types of experimental micro-nests containing 30 workers (Fig. 1): without reproductives (R−), with reproductives (R+), with aqueous polar extracts of workers (WE) or with aqueous polar extracts of reproductives (RE). These four combinations were set up using workers and brachypterous reproductives (i.e., secondary reproductives from nymphal lines^46^) originating from the same 10 colonies. Micro-nests were made with plastic boxes (50 mm diameter; Star-pack Cat#04913) with a cellulosic ultrapure paper (47 mm diameter; Whatman, grade 42 Ashless) humidified with 250 µl of microfiltered water and 0.5 g of Fontainebleau sand (Carlo Erba Reagents, granulometry: 180–500 µm).

For the R+ treatments, reproductives were sexed on a CO_2_ pad and then randomly paired (one male with one female) in the corresponding micro-nests. For R− and R+ treatments, 95 µL of ultrapure water was deposited on the sand of each micro-nest, whereas for extract treatments (WE and RE), 95 µL of polar extracts made from either workers or reproductives originated from the same colonies (i.e., each extract was done on the same colony as the tested workers; see details in the “polar extracts” section) were deposited on the sand. Note that the extracts were deposited to the sand contained in an Eppendorf tube. After the deposit of the extract, the sand samples were dropped off into the micro-nest, then the reproductives were added if any, and at the end, the 30 workers were added to the micro-nest (Fig. 1).

Video recordings were processed for each micro-nest and treatment for the 10 colonies to assess body-shaking behavior of workers in a room at 26 ± 1 °C with 55 ± 5% of relative humidity. Each micro-nest was transferred to a video recording chamber where individuals could settle down for 5 min before being distributed in groups according to treatments and recorded for 5 min (Panasonic HC-VXF990) under infrared lights. Two series of recordings were done 1 h after the micro-nest setup and 24 h later. Videos were recorded in random order under double-blind conditions^47^. The total number of body-shaking events expressed by all the workers during the 5 min of recordings were analyzed using the BORIS freeware v.6.0.5^48^. Note that a body-shaking event lasts on average 0.7 s, and the quantification of their occurrence was done according to previous publications^43,44^. At the beginning of each video recording, the humidity and temperature of the room were measured. At the end of the experiment, the mortality and sex ratio (number of males over females) of each box were calculated.

### Polar extracts

Workers and brachypterous reproductives used for polar extracts (WE and RE treatments respectively) were isolated from their stock colony and then sexed on a CO_2_ pad. For each colony (n = 10), a pool of workers (5 males and 5 females) was extracted for the WE treatments, and a pool of reproductives (1 male and 1 female) was extracted for the RE treatments. We used this ratio of 5 workers for 1 reproductive to reduce quantitative differences during extractions due to body size differences between workers and reproductives, and so to maximize behavioral responses during experiments. Indeed, based on fresh body mean weight (n = 15 colonies), one reproductive (7.93 ± 0.25 mg) is equivalent to 2.88 workers (2.75 ± 0.15 mg). According to preliminary tests and literature data^38^, ultrapure water was selected as the best polar solvent for the extraction of CPCs. Individuals were washed alive in 100 µL of ultrapure water with a vortexing period of 10 s (LP vortex mixer, 1200 rpm) in a 650 µL plastic Eppendorf. Individuals were incubated at room temperature (20 °C) for 5 min. At the end of the incubation time, an additional 10 s of vortexing was done before transferring the extracts into new vials. Over the 100 µL of polar extracts, 95 µL were used for behavioral experiments and 5 µL were analyzed by spectrophotometry for quality control. Three µL of each extract were measured by spectrophotometry (Varian Cary 50-scan®) at 215 nm (with 3 consecutive readings). Extracts with optical density values lower than the mean value of the water used as control were replaced.

### Mass spectrometry analyses

#### Compound profiling by MALDI-TOF MS

The protein profile of the cuticular polar extracts was analyzed using MALDI-TOF mass spectrometry for workers and brachypterous reproductives of each sex. The protocol described above in the “polar extracts” section was used to extract compounds, except that extractions were done on single individuals and not on a pool of individuals, per caste and sex for the 15 different colonies. An analysis of protein content in extracts was done using spectrophotometric protein quantification (as described above) and MALDI-TOF MS analysis on 5 individual extracts per sex and caste. These analyses showed that worker extracts present a lower concentration of compounds compared to reproductives. To limit detection bias in mass spectrometry by leveling up concentrations between workers and reproductives, for subsequent analyses 10 individual extracts of worker per sex were pooled for each colony (n = 10 pools per sex). Since statistical analysis of the worker samples either from individuals (n = 5 per sex) or from pools (n = 10 per sex) did not present significant differences, both datasets were treated as a single dataset. For reproductives, as mentioned above, analyses were always done individually (n = 15 per sex).

The 100 µL of cuticular extracts were stored at − 80 °C until treatment for MALDI-TOF MS analysis. Samples were vacuum concentrated 20-fold using a SpeedVac system (Thermo Scientific). The matrix solution and the MALDI deposition method were chosen to optimize the detection of peptides and proteins. Concentrated samples were diluted 5-fold in a solution of 4-hydroxy-α-cyano-cinnamic acid saturated in a solution of 33.3% acetonitrile, 66.6% water, and 0.1% trifluoroacetic acid. Matrix-sample solutions were spotted onto a gold-plated sample probe using the ultrathin layer method^49,50^. The excess liquid was vacuum-aspirated, and the spots were washed with cold 0.1% TFA to remove contaminants. MALDI-TOF–MS spectra were acquired on an UltraFlex I or an UltrafleXtreme mass spectrometer (Bruker Daltonics) in the linear positive mode in the 1000 to 25000 *m/z* range. Calibrations were performed externally using apomyoglobin and cytochrome c ion peaks, using the near-neighbor spot method. MALDI-TOF–MS spectra were processed using FlexAnalysis 3.3 software (Bruker). Spectra were smoothed using the gaussian algorithm with a width of 1 Da and 5 cycles. Peaks were annotated using the Sum algorithm with a S/N ≥ 4 and a peak width of 5 Da.

For protein/peptide profile comparisons, spectra were exported from the FlexAnalysis software into the mMass open-source software v5.5.0 (Strohalm et al. 2008) for further treatment. Spectra were averaged when necessary and negative spectra were generated by subtraction from zero.

#### Identification of proteins/petides by top-down MS/MS sequencing

To confirm that the profiled extracted cuticular compounds were proteins and/or peptides, top-down MS sequencing of species was performed using MALDI-TOF/TOF LIFT or ESI-QTOF CID fragmentation method. For MALDI-TOF/TOF LIFT, the same sample preparation and deposition method as for the profiling approach were used. For ESI-QTOF CID analysis, samples were diluted in the micromolar range in a solution of 50% acetonitrile, 49.8% water and 0.2% formic acid and were analyzed by direct infusion in a 4-GHz MaXis ultra-high-resolution quadrupole-TOF mass spectrometer (Bruker Daltonics) equipped with an electrospray ion source. Spectra were acquired in positive ion MS mode over a 115–3000 *m/z* range and were externally calibrated with the ESI-L Low Concentration Tuning Mix (Agilent Technologies). ESI mass spectra were processed using DataAnalysis v3.1 software (Bruker Daltonics). The PEAKS software was used for de novo sequencing of the compounds and spectra interpreted manually. To identify proteins based on the de novo sequence tags, we used the UniProt Peptide search tool to interrogate the TrEMBL and SwissProt Eukaryotes databases. MS/MS data were exploited using a Mascot search, performed from top-down spectra in the two databases: *Termitoidae* protein database and *Termitoidae* nucleotide database.

### Statistical analyses

The number of body-shaking events counted on each video recording was analyzed using a general linear mixed model (LMM) with several explanatory variables: treatments, time, sex-ratios of workers, mortality, temperature, and humidity. Non-significant variables and interactions were removed following a step-by-step procedure which include sex-ratios, mortality, temperature, and humidity. Note that the average temperature and humidity during video recordings were respectively 25.59 °C ± 0.01 (mean ± SEM) and 57.50%RH ± 0.12. No worker mortality was observed after 1 h, and 3 out of 1200 workers were dead after 24 h. The sex-ratio (male over female) were on average 1.47 ± 0.07. Therefore, the explanatory factors used in the final model were the treatments and the time (with their interactions). To fit with homoscedasticity and normal distribution of model residuals, the numbers of body-shaking were log-transformed. Colonies were included as a random factor in all statistical models. To explore the effects of treatments and time separetly (no-significant interaction were observed), the dataset was split and two additional LMMs were conducted on either 1 h or 24 h with treatments as an explanatory factor. Post-hoc pairwise comparisons were conducted using Tuckey HSD. Time effect comparisons (i.e., 1 h vs 24 h) was analyzed for each treatment separately with non-parametric Wilcoxon signed-rank tests. Outliers were calculated with the default interquartile range criterion of 1.5.

For each caste and sex sample, MALDI-TOF mass spectra were treated in FlexAnalysis as described above to calculate peak areas. Peaks with a standard deviation below 1 Da were assigned to the same compound up to *m/z* 9800. For *m/z* > 9800, a standard deviation less than 1.7 Da was used as threshold due to the lower resolution and intensity of the peaks. Compounds that were observed only once among the samples were not considered and deleted from the mass list. Although some peaks may correspond to doubly charge species, all *m/z* values were treated as separate species in statistical analyses. Then peak areas were analyzed using two multivariate analyses. Centered and autoscaled data were tested with and without quadratic transformation. Since no improvement was shown with transformation, further analyses were done without^51^. To discriminate groups based on their protein profiles, Partial Least Squares Discriminant Analyses (PLS-DA) were done on peak areas with the plsda function (*mixOmixs* package) on 4 pre-defined groups (female reproductives, male reproductives, female workers, and male workers). Then VIP values were calculated to determine the importance of each CPC in the projection. The most representative CPCs with a VIP value greater than 1 were thereafter noted as major compounds^52^. Comparisons between sexes within each caste were done on the relative peak areas (calculated by dividing a peak area by the total area of all the peaks in the spectrum) with non-parametric Wilcoxon signed-rank tests.

All statistical analyses were conducted using the software R v4.1.2 (www.r-ptoject.org) loaded with the packages *lme4*, *emmeans*, *car*, *multcompView*, *Hotelling*, *ade4*, *mixOmics, RVAideMemoire*, and *ggplot2*. Multiple comparisons were corrected with FDR as a *p*-value adjustement method.

## Results

### Behavioral experiments

The number of body-shaking events was influenced significantly by the treatment factor within each time point (1 h or 24 h). Therefore, the dataset was split and analyzed separately for each time point. Indeed, the number of body-shaking events was dependent on the time after the setup of the micro-nests (F_(3,17.6)_ = 31.66, *p* = 0.002) with no interaction with the treatments (F_(3,17.6)_ = 1.62, *p* = 0.18).

**Figure 2:**
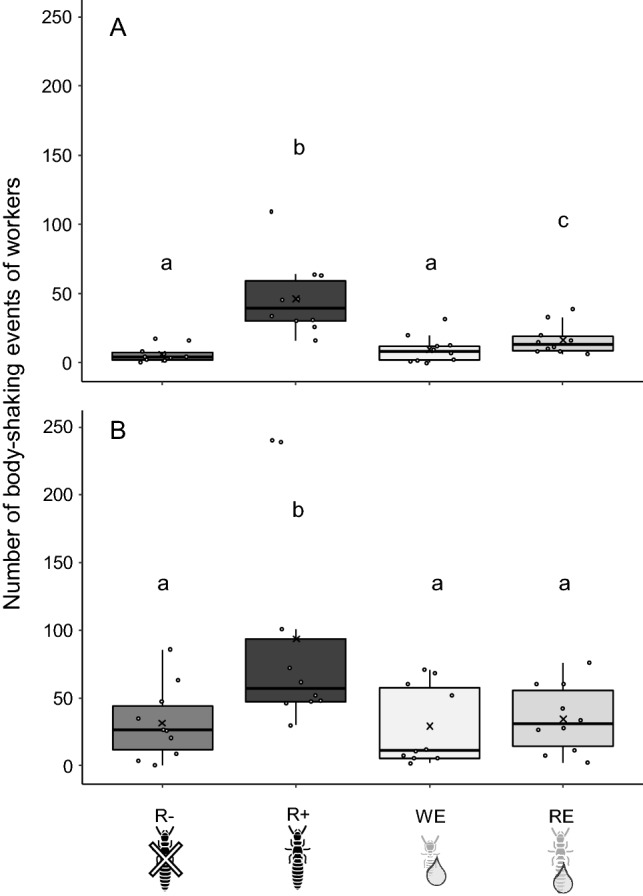
Number of body-shaking events of workers after 1 h (**A**) or 24 h (**B**) for the different treatments (n = 10). In absence of reproductives (R−), in the presence of reproductives (R+), with extracts of workers (WE) or reproductives (RE). Box plots show the median, upper and lower quartiles, and range; dots, outliers; crosses, means; different letters indicate significant differences (LMMs, α = 0.05).

At 1 h, there was a significant effect of the treatments on the number of body-shaking (F_(3,17.6)_ = 32.47, *p* < 0.001; Fig. 2A). Post-hoc tests showed that workers displayed more body-shaking events when exposed to live reproductives or their extracts (R+: 46.4 ± 8.5 ; RE: 16.6 ± 3.5 ; values are mean ± SEM) than when exposed to a workers-only environment (R−: 6.1 ± 1.9) (R+ vs R−: t_(27)_ = 8.96, *p* < 0.001; RE vs R−: t_(27)_ = 4.61, *p* < 0.001) or than when exposed to extracts from workers (WE: 9.7 ± 3.2) (R+ vs WE: t_(27)_ = 7.82, *p* < 0.001; RE vs WE: t_(27)_ = 3.47, *p* = 0.009). Worker's extract did not significantly influence the number of body-shaking (WE vs R−: t_(27)_ = 1.14, *p* = 0.668).

At 24 h after the setup of the micro-nests (Fig. 2B), the effect of the treatments was still significant (F_(3,22.8)_ = 6.65, *p* < 0.001). However, post-hoc tests showed that only the presence of live reproductives (R+: 93.7 ± 8.5) was significantly different from the other treatments (R+ vs R−: 31.6 ± 8.6, t_(27)_ = 3.67, *p* = 0.006; R+ vs WE: 29.3 ± 9.3, t_(27)_ = 4.00, *p* = 0.002; R+ vs RE: 34.6 ± 7.9, t_(27)_ = 2.96, *p* = 0.030).

**Figure 3:**
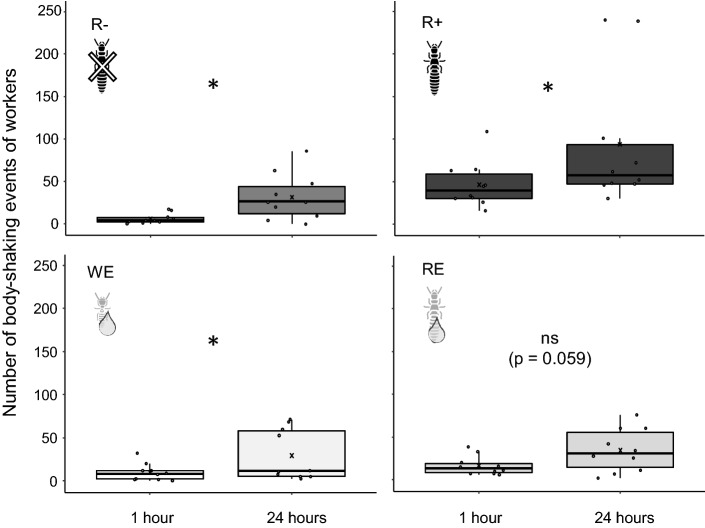
Number of body-shaking events of workers after 1 h and 24 h for the different treatments (n = 10). In absence of reproductives (R−), in the presence of reproductives (R+), with extracts of workers (WE) or reproductives (RE). Box plots show the median, upper and lower quartiles, and range; dots, outliers; crosses, means; * indicates significant differences (Wilcoxon signed-rank test, *p* < 0.05).

Direct comparisons of the number of body-shaking events at 1 h and 24 h for each treatment (Fig. 3) showed a time-dependent increase of events for 3 treatments: with a workers-only environment (R−: Z = 4.5, *p* = 0.022), with live reproductives (R+: Z = 6, *p* = 0.032), and with extracts from workers (WE: Z = 0.5, *p* = 0.022). Indeed, body-shaking events increased over time only marginally significantly in the presence of extracts from reproductives (RE: Z = 8.5, *p* = 0.059).

### Protein/peptide profiling by MALDI-TOF MS

**Figure 4:**
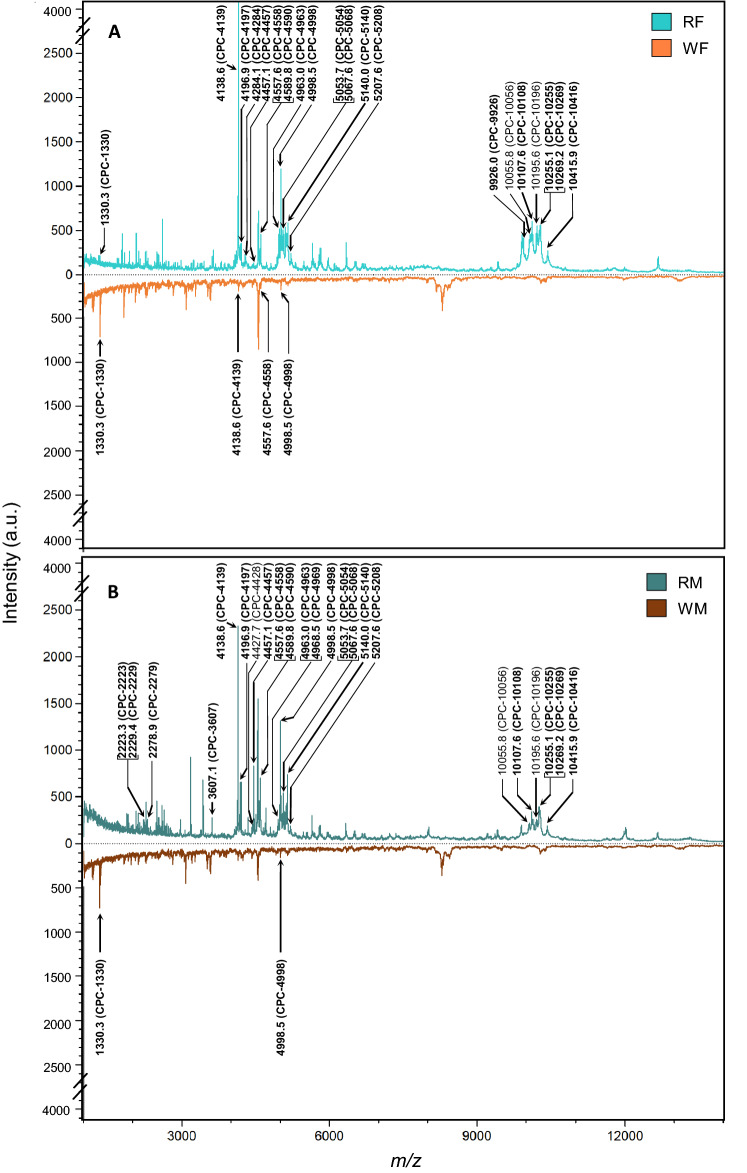
Averaged mass spectra of cuticular polar extracts from MALDI-TOF analyses. Spectra are shown in positive/negative display for reproductives versus workers for both (**A**) females and (**B**) males. The spectra are based on a sum up of 15 spectra for each caste and sex. Annotated peaks represented in bold are the first-quartile of the 87 major CPCs identified as discriminant by the PLS-DA analyses with VIP values greater than 1. Note that the 3 major CPCs 4428, 10056 and 10196 are also represented in the figure to support the discussion even if they are not in the first-quartile (see the result section for details). See Table 1 for the entire list of compounds.

**Figure 5:**
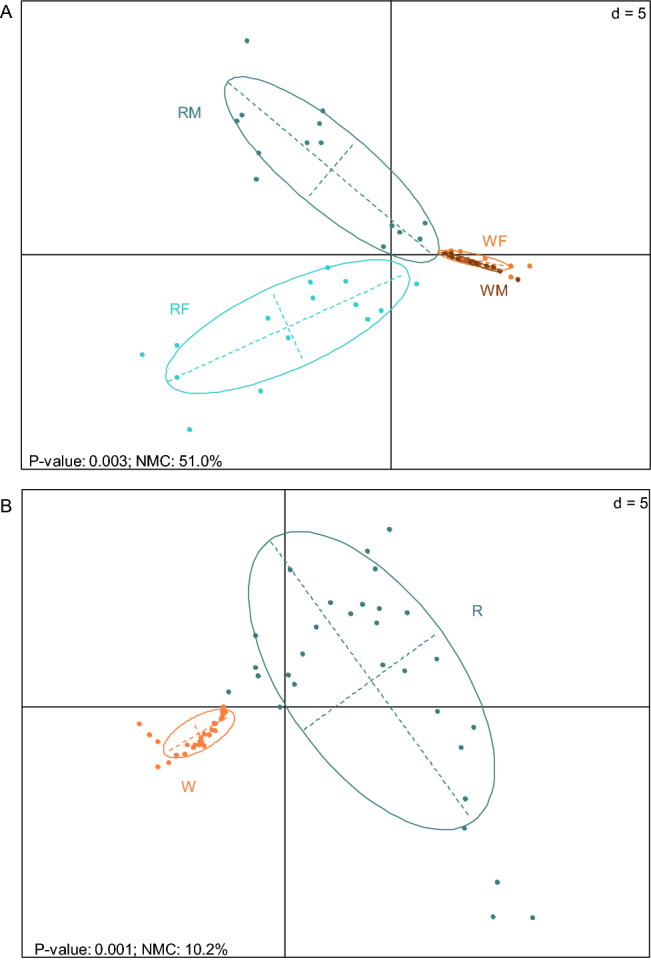
PLS-DA plot based on the peak areas of the 197 CPCs identified from cuticular polar extracts (n = 15 per group; peaks were selected using the Sum algorithm with a S/N ≥ 4 and a peak width of 5 Da). (**A**) Score plot of female reproductives (RF, light turquoise), male reproductives (RM, dark green), female workers (WF, light orange), and male workers (WM, dark orange). (**B**) Score plot of reproductives (dark green) and workers (light orange). Score plot display centroids and dispersion ellipses (cellipse = 1.5).

Analyses performed on cuticular polar extracts of reproductives and workers (females and males), using a MALDI-TOF MS method designed for protein detection, revealed 197 CPCs ranging from *m/z* 1000 to 25000 (Fig. 4). PLS-DA analyses based on the peak areas of CPCs were performed to provide a visual assessment of the repartition of individuals according to caste and sexe (Fig. 5). PLS-DA discriminated extracts according to their caste only with a Number of MissClassification (NMC) of 51.0% (Fig. 5A). The Number of MissClassification drops to 28.5% if workers of both sexes are pooled together, and to 10.2% if individuals are regrouped according to their reproductive status whatever their sex (Fig. 5B). In a permutation test with 999 repetitions, the MVA test was statistically significant (all groups *p* = 0.003; caste only *p* = 0.001). Pairwise comparisons using permutation tests based on cross model validation, separated reproductives (RF and RM) from workers (WF and WM) independently of their sexes (*p* = 0.001).

According to VIP values, there are 87 major CPCs ranging from *m/z* 1000 to 25000 (Table 1). Profile comparisons of the 87 major CPCs of the 4 groups (RF, RM, WF, WM) revealed that 85 CPCs were specific to reproductives and 2 CPCs were specific to workers. Among all 7 CPCs detected in workers (i.e., CPCs specific to workers or CPCs shared with reproductives), 5 were common to females and males, and 2 were specific to females. Among the 85 CPCs detected in the reproductives, 48 were common to female and male reproductives, 19 were female-specific, and 18 male-specific. The mean relative areas of the major compounds common to both sexes were compared within each caste. These comparisons revealed that three compounds present higher mean relative areas in female reproductives compared to male reproductives: CPC-4963 (W = 153, *p* = 0.0252), CPC-10196 (W = 156, *p* = 0.022), and CPC-10416 (W = 166, *p* = 0.013). No significant sexe differences where found for workers.

**Table 1:** Compounds identified from the MALDI-TOF analyses for female reproductives (RF), male reproductives (RM), female workers (WF), and male workers (WM). CPCs average *m/z* values are also reported with their presence/absence in each group.

CPC	*m/z* (average)	VIP	RF	RM	WF	WM
CPC-1001	1001,0	0,530436			X	X
**CPC-1020**	**1019,6**	**1,021573**	**X**	**X**	**X**	**X**
CPC-1025	1024,8	0,494077		X	X	
CPC-1185	1184,6	0,498058			X	
**CPC-1321**	**1321,5**	**1,004338**	**X**			
**CPC-1330**	**1330,3**	**1,469192**	**X**		**X**	**X**
CPC-1671	1671,2	0,604704	X	X		
CPC-1699	1698,6	0,849026	X	X		
CPC-1740	1740,3	0,57284	X	X		
CPC-1778	1778,3	0,989368	X			
CPC-1818	1817,9	0,510739			X	
**CPC-1826**	**1826,5**	**1,11122**	**X**			
**CPC-1885**	**1885,4**	**1,063081**	**X**	**X**		
CPC-1910	1909,6	0,150182	X	X	X	
CPC-1915	1915,1	0,758832		X	X	
CPC-1971	1970,9	0,521422			X	X
CPC-1988	1988,0	0,703959	X			X
CPC-2052	2051,9	0,535768			X	
**CPC-2070**	**2070,1**	**1,309227**	**X**	**X**		
CPC-2094	2093,7	0,531649	X	X		
CPC-2111	2111,4	0,516531			X	X
CPC-2115	2115,4	0,63919	X	X		
CPC-2144	2144,2	0,646186	X	X		
**CPC-2181**	**2180,6**	**1,174229**		**X**		
**CPC-2223**	**2223,3**	**1,526415**		**X**		
**CPC-2229**	**2229,4**	**1,397133**		**X**		
CPC-2258	2257,8	0,576948	X	X	X	X
CPC-2268	2267,9	0,490154			X	X
**CPC-2279**	**2278,9**	**1,559966**		**X**		
CPC-2286	2286,2	0,662056			X	X
CPC-2296	2295,7	0,876546	X	X		
CPC-2464	2463,7	0,534513			X	X
CPC-2481	2481,3	0,967852	X	X		
CPC-2509	2509,3	0,898811	X	X		
CPC-2521	2520,6	0,534598			X	X
**CPC-2526**	**2525,8**	**1,085698**		**X**		
CPC-2556	2555,6	0,546227	X	X		
**CPC-2571**	**2570,6**	**1,087777**	**X**	**X**		
**CPC-2595**	**2595,4**	**1,015188**	**X**	**X**		
**CPC-2647**	**2647,0**	**1,127627**	**X**	**X**		
CPC-2672	2672,4	0,713963	X	X		
CPC-2718	2718,5	0,060407	X	X	X	X
**CPC-2761**	**2761,2**	**1,276871**		**X**		
CPC-2813	2812,9	0,536367			X	X
CPC-2973	2973,3	0,742952	X	X		
CPC-3076	3076,5	0,848633			X	X
CPC-3126	3125,8	0,531143	X	X		
**CPC-3175**	**3175,5**	**1,024943**	**X**	**X**		
CPC-3204	3204,1	0,518373			X	X
CPC-3229	3228,8	0,726335	X	X		
CPC-3268	3267,6	0,747726			X	X
CPC-3385	3384,9	0,620502	X	X		
CPC-3429	3428,7	0,987307		X		
CPC-3520	3520,1	0,911821			X	X
**CPC-3574**	**3573,6**	**1,041259**			**X**	**X**
**CPC-3607**	**3607,1**	**1,120084**		**X**		
CPC-3672	3672,2	0,917646		X		
CPC-3792	3791,5	0,73626	X	X		
CPC-3854	3854,4	0,598058	X	X		
**CPC-4058**	**4058,5**	**1,095836**	**X**			
CPC-4082	4082,3	0,885966	X	X		
**CPC-4094**	**4094,3**	**1,334512**	**X**	**X**		
**CPC-4122**	**4122,3**	**1,186635**	**X**	**X**		
**CPC-4139**	**4138,6**	**1,509201**	**X**	**X**	**X**	
CPC-4153	4153,4	0,905559	X	X		
CPC-4160	4159,6	0,629695	X		X	
**CPC-4170**	**4170,3**	**1,214589**	**X**	**X**		
**CPC-4180**	**4179,7**	**1,173423**	**X**			
CPC-4187	4186,5	0,624585	X	X		
CPC-4189	4188,6	0,792427	X	X		
**CPC-4197**	**4196,9**	**1,708456**	**X**	**X**		
CPC-4248	4247,5	0,161449	X	X	X	
CPC-4255	4255,0	0,529883	X	X		
**CPC-4270**	**4270,2**	**1,246074**	**X**			
**CPC-4281**	**4281,5**	**1,297332**		**X**		
**CPC-4284**	**4284,1**	**1,411651**	**X**			
CPC-4317	4317,3	0,590326		X	X	
**CPC-4345**	**4344,9**	**1,100082**	**X**	**X**		
CPC-4423	4423,3	0,516437			X	X
**CPC-4428**	**4427,7**	**1,185141**		**X**		
**CPC-4457**	**4457,1**	**1,681298**	**X**	**X**		
**CPC-4499**	**4499,3**	**1,111521**		**X**		
**CPC-4527**	**4526,6**	**1,267799**		**X**		
**CPC-4558**	**4557,6**	**1,493011**	**X**	**X**	**X**	
CPC-4573	4573,8	0,71769	X	X		
**CPC-4574**	**4574,3**	**1,27198**	**X**	**X**		
**CPC-4590**	**4589,8**	**1,85957**	**X**	**X**		
**CPC-4614**	**4613,6**	**1,276005**		**X**		
**CPC-4660**	**4660,4**	**1,260221**		**X**		
**CPC-4700**	**4699,9**	**1,262092**	**X**	**X**		
CPC-4705	4705,1	0,543172	X	X		
**CPC-4801**	**4800,9**	**1,003368**	**X**	**X**		
**CPC-4950**	**4949,6**	**1,11043**	**X**	**X**		
**CPC-4963**	**4963,0**	**1,517741**	**X**	**X**		
**CPC-4969**	**4968,5**	**1,406685**		**X**		
CPC-4981	4981,3	0,91088	X			
CPC-4993	4993,1	0,628464	X	X		
**CPC-4998**	**4998,5**	**1,536198**	**X**	**X**	**X**	**X**
**CPC-5014**	**5014,4**	**1,341084**	**X**	**X**		
CPC-5017	5017,4	0,649505	X	X		
CPC-5020	5020,0	0,967497	X			
CPC-5028	5027,6	0,922054	X	X		
**CPC-5041**	**5041,4**	**1,388364**	**X**	**X**		
**CPC-5054**	**5053,7**	**1,658253**	**X**	**X**		
CPC-5061	5060,8	0,944273	X	X		
**CPC-5068**	**5067,6**	**1,589932**	**X**	**X**		
**CPC-5076**	**5076,2**	**1,123753**	**X**			
CPC-5098	5097,7	0,754754	X	X		
**CPC-5101**	**5100,9**	**1,004803**	**X**	**X**		
CPC-5105	5105,1	0,672846	X	X		
CPC-5110	5109,7	0,898385	X	X		
**CPC-5119**	**5119,5**	**1,16972**	**X**			
**CPC-5127**	**5126,9**	**1,334117**	**X**	**X**		
**CPC-5130**	**5130,2**	**1,382131**		**X**		
CPC-5133	5133,0	0,756936	X	X		
CPC-5135	5135,2	0,999309	X	X		
**CPC-5140**	**5140,0**	**1,445892**	**X**	**X**		
CPC-5143	5142,6	0,59222	X	X		
CPC-5156	5155,7	0,888511	X	X		
CPC-5195	5194,5	0,555855	X	X		
**CPC-5208**	**5207,6**	**1,390999**	**X**	**X**		
**CPC-5273**	**5272,7**	**1,176503**	**X**			
CPC-5303	5303,4	0,535279	X	X		
CPC-5466	5466,4	0,858169	X	X		
CPC-5539	5539,5	0,811476	X	X		
**CPC-5628**	**5628,3**	**1,102437**	**X**			
**CPC-5642**	**5642,1**	**1,149421**	**X**	**X**		
CPC-5658	5657,7	0,679231	X	X		
**CPC-5686**	**5686,0**	**1,273353**		**X**		
**CPC-5702**	**5701,9**	**1,114943**	**X**			
**CPC-5774**	**5774,4**	**1,022658**	**X**	**X**		
**CPC-5788**	**5788,4**	**1,007828**	**X**	**X**		
**CPC-5804**	**5804,0**	**1,179976**	**X**	**X**		
**CPC-5819**	**5819,3**	**1,020338**	**X**			
CPC-5832	5831,5	0,624797	X	X		
CPC-5950	5950,2	0,630534	X	X		
**CPC-5964**	**5964,4**	**1,040088**	**X**	**X**		
CPC-6086	6086,2	0,786967	X	X		
CPC-6332	6331,7	0,840422	X	X		
**CPC-6346**	**6345,7**	**1,044058**	**X**	**X**		
CPC-6492	6492,4	0,786359	X	X		
CPC-6508	6507,8	0,964391	X	X		
CPC-6662	6662,1	0,700461	X	X		
CPC-7218	7218,1	0,547197	X	X		
CPC-7543	7542,8	0,526836	X	X		
CPC-7643	7642,8	0,613764	X	X		
CPC-7973	7973,0	0,661666			X	X
**CPC-8008**	**8007,7**	**1,163923**		**X**		
CPC-8164	8163,8	0,801151			X	X
CPC-8232	8232,4	0,536915			X	X
CPC-8264	8263,6	0,518308				X
**CPC-8276**	**8275,7**	**1,010273**			**X**	**X**
CPC-8277	8277,5	0,802915			X	X
CPC-8309	8308,9	0,803213			X	X
CPC-8391	8390,7	0,654338			X	X
CPC-8433	8432,6	0,761802			X	X
CPC-9270	9269,9	0,967526	X	X		
**CPC-9408**	**9407,7**	**1,164563**	**X**			
CPC-9411	9410,6	0,810851	X	X		
CPC-9844	9844,3	0,666565	X	X		
**CPC-9899**	**9899,1**	**1,168643**	**X**	**X**		
**CPC-9926**	**9926,0**	**1,443105**	**X**			
CPC-9960	9959,9	0,889133	X	X		
CPC-9965	9964,6	0,544774	X	X		
**CPC-10056**	**10055,8**	**1,056447**	**X**	**X**		
**CPC-10060**	**10059,8**	**1,128508**	**X**	**X**		
**CPC-10064**	**10064,4**	**1,116155**	**X**			
**CPC-10083**	**10083,0**	**1,371353**	**X**			
CPC-10106	10105,9	0,83495	X	X		
**CPC-10108**	**10107,6**	**1,483708**	**X**	**X**		
CPC-10122	10121,7	0,953588	X	X		
CPC-10135	10134,7	0,560386	X	X		
**CPC-10138**	**10137,6**	**1,21449**	**X**	**X**		
**CPC-10190**	**10190,2**	**1,132488**	**X**			
**CPC-10196**	**10195,6**	**1,030351**	**X**	**X**		
CPC-10199	10198,5	0,830378	X	X		
**CPC-10209**	**10209,2**	**1,031542**	**X**	**X**		
CPC-10232	10232,4	0,899468	X	X		
**CPC-10252**	**10252,2**	**1,232356**		**X**		
**CPC-10255**	**10255,1**	**1,489401**	**X**	**X**		
**CPC-10269**	**10269,2**	**1,977359**	**X**	**X**		
CPC-10283	10282,6	0,864993	X	X		X
CPC-10285	10285,0	0,585131	X	X		
CPC-10300	10300,2	0,608234	X	X		
CPC-10311	10310,8	0,597246	X	X		
CPC-10381	10381,0	0,627671	X	X		
CPC-10383	10383,4	0,531412	X	X		
CPC-10394	10394,4	0,536455	X	X		
CPC-10402	10401,8	0,769565	X	X		
**CPC-10413**	**10412,8**	**1,221522**	**X**	**X**		
**CPC-10416**	**10415,9**	**1,937273**	**X**	**X**		
CPC-10419	10418,8	0,577654	X	X		
**CPC-10781**	**10781,2**	**1,072369**	**X**			
CPC-11980	11979,6	0,790936	X	X		
**CPC-12012**	**12012,4**	**1,064564**	**X**	**X**		
CPC-12662	12661,7	0,612341	X	X		
CPC-12667	12667,0	0,599706	X	X		

**Figure 6:**
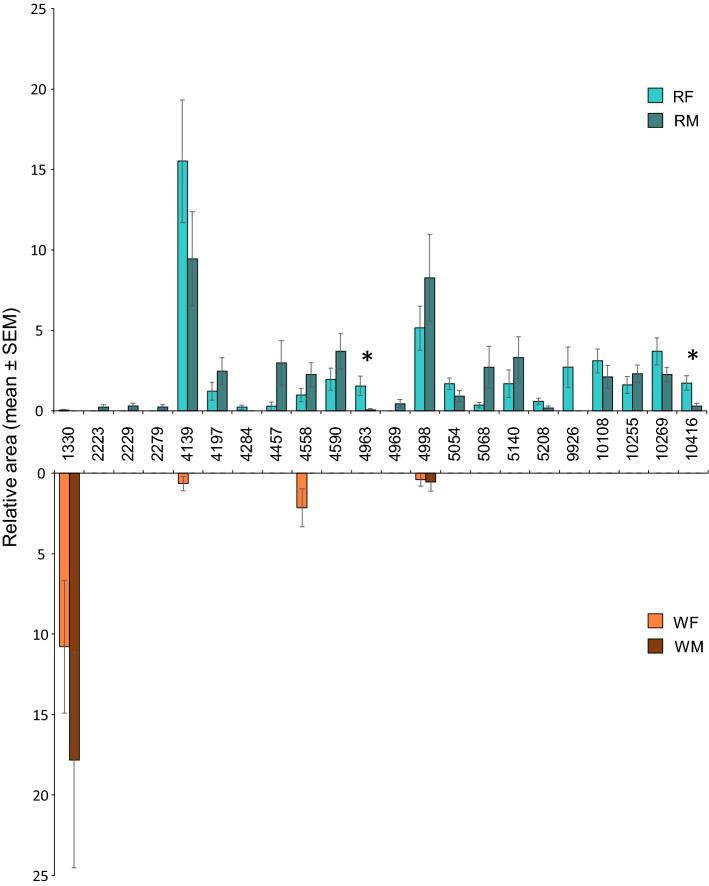
Relative areas of the first-quartile of the 87 major CPCs identified as discriminant by PLS-DA analyses for reproductives (**A**) and workers (**B**) of both sexes: female reproductives (RF), male reproductives (RM), female workers (WF) and male workers (MW). Relative areas are represented as mean (± SEM) and are calculated on all the spectra (see Table 1 for CPCs numbers). * indicates significant differences (Wilcoxon signed-rank test, *p* < 0.05; Multiple comparisons were corrected with FDR as the *p*-value adjustement method).

The VIP column represents the VIP score obtained for each compound (see the method section for details). The CPCs in bold are the 87 major ones, identified as discriminant by the PLS-DA analyses with VIP values greater than 1. Underlined are the first-quartile of the 87 major CPCs (also represented in Fig. 6 and Sup. Figure 1).

Over the first quartile of the 87 majors CPCs (Fig. 6), CPC-4139 presents the highest mean relative area in reproductives, whereas CPC-1330 is the highest peak in workers (see the full list in Table 1). Interestingly, two CPCs are specific to female reproductives (CPC-4284 and CPC-9926) and four CPCs are specific to male reproductives (CPC-223, CPC-2229, CPC-2279, CPC-4969). A visual comparison of the compounds repartition across castes and sexes of the first-quatile of the 87 major CPCs is shown in the supplementary Fig. 1 (CPCs were attributed to a group if more than one individual present the compound). Overall, in *R. flavipes* the diversity in workers' proteins is very low compared to reproductives (Sup. Figure 2).

### Identification of protein/peptide components by top-down MS sequencing

**Figure 7:**
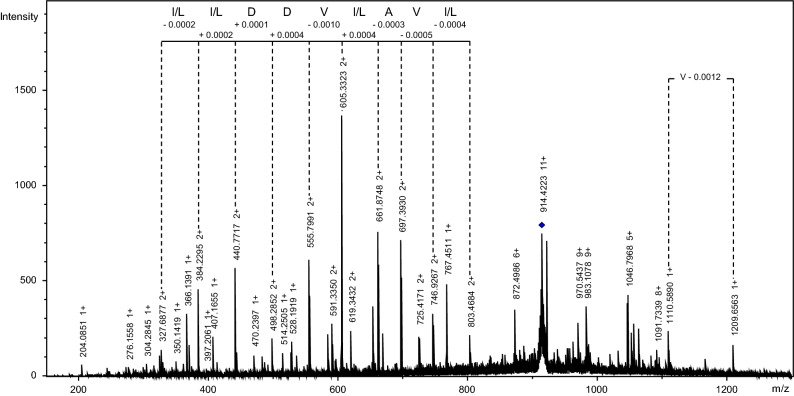
Fragmentation mass spectrum of the CPC-10056 using UHR-ESI-QTOF-MS/MS CID. The precursor ion *m/z* 914.4 (11+) is represented with a blue lozenge.

To confirm that the profiled cuticular extracts correspond to proteins and/or peptides, we performed mass spectrometry fragmentation on CPC-4428 and CPC-10056. Clear fragmentation spectra were produced for these two VIP CPCs. Manual interpretation of MS/MS spectra showed that inter-peak distances correspond to amino acids, as shown for CPC-10056 (Fig. 7).

Further interpretation of the spectra allowed for the determination of sequence tags for these two CPCs (Table 2). Search with the UniProt Peptide search tool in the TrEMBL and SwissProt protein databases for the sequence tag from CPC-10056 (I/LI/LDDVI/LAVI/L) generated seven hits in Eukaryotes (Table 3). Within the *Insecta* taxon, the protein G12 precursor from *Culex quinquefasciatus* (southern house mosquito) was found under the B0WED5 Uniprot entry, while two uncharacterized proteins from *Coptotermes formosanus* (Formosan subterranean termite) were found under the A0A6L2P7Q1 and A0A6L2PEY0 entries. A Mascot search in the NCBI databases on MS/MS data for CPC-10056 (*m/z* 10055.8) identified the JK369675.1 sequence from *Coptotermes formosanus* in the *Termitoidae* cDNA library, with a score of 81 (Table 4). This hit contains the manually annotated de novo tag I/LI/LDDVI/LAVI/L from CPC-10056.

**Table 2:** Sequence tags obtained by de novo sequencing for CPC-4428 and CPC-10056.

Compound	m/z (average)	Precursor		Corresponding monoisotopic mass M (Da)	Fragmentation method	Sequence tag
		*m*/*z* (monoisotopic)	z			
CPC-4428	4427.7	4425.263	1	4424.255	LIFT	A E Y P I/L
CPC-10056	10055.8	914.4171	11	10047,501	CID	I/L I/L D D V I/L A V I/L

**Table 3:** Eukaryota hits identified with the sequence tag I/LI/LDDVI/LAVI/L.

Entry	*Protein name*	Taxon
A0A371IC81	Protein GIGANTEA	Viridiplantae/Mucuna pruriens
A0A1U8QM93	ADSL_C domain-containing protein	Fungi/Aspergillus nidulans
Q5B619	ADSL_C domain-containing protein	Fungi/Aspergillus nidulans
A0A5E3WGF4	RNA-directed RNA polymerase	Fungi/Peniophora
A0A6L2P7Q1	Uncharacterized protein	Metazoa/Insecta/Termitoidae/Coptotermes
A0A6L2PEY0	Uncharacterized protein	Metazoa/Insecta/Termitoidae/Coptotermes
B0WED5	G12	Metazoa/Insecta/Culex

The Peptide Search tool from Uniprot was used to interrogate the SwissProt and TrEMBL databases. Isoleucine and Leucine are treated as equivalent by Peptide Search.

**Table 4:** Results of protein- and nucleotide- sequence based TDS MASCOT search in *Termitoidae* NCBI databases. All matches with Mascot score lower than 53 were considered insignificant.

Compounds	*m/z* (average)	Precursor		Corresponding monoisotopic mass M (Da)	Fragmentation method	Protein database hit	Nucleotide database hit
		*m/z* (monoisotopic)	z				
CPC-4428	4427.7	4425.263	1	4424.255	LIFT	No significant match	No significant match
CPC-10056	10055.8	914.4171	11	10047.501	CID	No significant match	JK369675.1 from *Coptotermes formosanus *metatranscriptomic cDNA library, score = 81

BLAST alignements on the sequence of the G12 precursor from *Culex quinquefasciatus* and the JK369675.1 sequence from *Coptotermes formosanus* showed similarities varying from ~ 39% to ~ 72% with protein G12 from termite species *Cryptotermes secundus* and *Zootermopsis nevadensis*. The protein G12 precursor from *Culex quinquefasciatus*, together with its homolog AEG12 induced in the midgut of adult female mosquitoes (*Aedes aegypti*) after a blood meal, is thought to have originated from a common ancestor and share a close similarity to a digestion-related cockroach allergen proteins also secreted in the midgut^53,54^. Altogether, these results suggest that CPC-10056 is an ortholog of G12 protein, which is present in cuticular polar extracts and specific to reproductives in our studied species *R. flavipes*.

## Discussion

In this preliminary work, we explore whether there is a potential basis for polar compounds, in particular proteins, to act on reproductive recognition in termites. For this purpose, we used aqueous extraction of polar compounds for both protein profiling and for behavioral assays. It has been previously demonstrated that body-shaking of workers (a vibratory behavior) was involved in reproductives recognition in the termite *R. flavipes*, since workers expressed this vibratory behavior in presence of reproductives or eggs^18,43,44^. Here we show that cuticular polar extracts from reproductives contain compounds that have the ability to trigger body-shaking, while extracts from workers do not. This effect is however lower than that observed with live termites. We also show that body-shaking behavior varies over time, with an increase in the number of events after 24 h, except for reproductive extracts. Using a MALDI-TOF MS method designed for protein detection, we have profiled proteins in the polar extracts of different castes and sexes, and obtained by ESI–MS/MS sequence tags on a sample of differentially observed peaks that confirm that the profiled compounds are essentially proteins. Henceforth, we have referred to the compounds detected in polar extracts using this method as Cuticular Protein Compounds (CPCs). Statistical analyses reveal a diverse mixture of CPCs, which is caste- and sex- dependent. Mass spectrometry fragmentation showed at least one example of an ortholog of G12, a digestive protein in termites which could be related to regurgitation by the insect. CPCs composition was more diverse in reproductives (171 CPCs in total) than workers (41 CPCs), with sex-specific CPCs for both male and female reproductives. The presence of a molecular signature of CPCs specific for a sex and/or caste raises the intriguing possibility that proteins could encode for social signals, as was shown for cuticular hydrocarbons (CHCs) or volatile compounds, which are well known to be involved in several social interactions^18–24^.

Chemical profiles with sex- or caste-specific compounds are well-known in numerous insect species, mainly supported in social insects by the cuticular hydrocarbons (CHCs)^25,55^. Indeed, CHCs are the most studied chemical cues since decades, and represent a key element of the division of labor in social insects. CHCs constitute a major part of the lipids covering the cuticle of insects, both in relative and absolute abundance^56^. The CHC composition vary between sexes and castes which has been shown to be a central factor involved in social interactions, social organization, recognition, sex/fertility signaling and mate choice, as well as in reproductive regulation in insects^22,25,56–58^, and more specifically in termites^21–23,42^. However, CHCs are not the only chemical components of insects’ cuticle^59^. While cuticular polar compounds diversity could encode information in addition to the well-known CHCs, their implication in social interactions has been largely neglected until now. The aqueous extracts prepared for the present study are not expected to contain non-polar compounds. However, we cannot rule out that at least part of the observed effect is due to polar compounds which are not proteins. Still, among polar compounds, proteins have physico-chemical characteristics that make them good candidates to hold non-volatile information, independently of or in concert with CHCs or non-polar compounds. Moreover, proteins are known to evolve to carry specific functions in organisms, and would be good candidates as specific mediators, whereas polysaccharides in the cuticle are likely to play mostly a structural role, with few variations between castes and sexes. In vertebrates and aquatic species, proteins are well-known to be major mediators for inter-individual interactions^3^. In insect societies, with structured nests, we could hypothesize that large biomolecules like CPCs could be deposited at nest’s hubs (royal chamber or nest entrance), allowing the transfer of contact information to walking nestmates, for example through gustatory perception. This is especially true for social insects living in close environments where contacts between individuals are numerous. Polar compounds such as CPCs could also be exchanged during social interactions like grooming or food transfer (social trophallaxis).

Globally in insects, investigations on proteins as signals are scarce and do not concern cuticular proteins^3^. Only few examples of proteins were described on the cuticle in bees, wasps and termites but no functional study was done^36–38^. In *Drosophila melanogaster*, the proteins contained in the seminal fluid are transferred to the female during mating, and they trigger various physiological and behavioral post-mating modifications of the female^60^. In honeybees, the proteins deposited by nurses in the royal jelly induce the development of larvae into future queens^61^. To our knowledge, the only studies investigating the behavioral role of proteins in termites showed the presence on the egg surface of an antibacterial lysozyme and a digestive β-glucosidase enzyme^62,63^. This protein complex called TERP (Termite Egg Recognition Pheromone) allow workers to recognize eggs. Here, we show evidence that CPCs could be as diverse and specific as CHCs. Indeed, the diversity and richness of reproductives' CPCs, and the behavioral response of workers exposed to polar extracts from reproductives, seems to point to their involvement in reproductive recognition. Whether CPCs are cues (i.e., unselected by-products of the metabolism) or signals (evolved for the benefit to both the sender and the receiver) remain to be determined^2^. Further work is needed to show if a set of CPCs induces behavioral answer in the subterranean termite *R. flavipes,* and to identify these proteins.

Reproductive signaling (or fertility signaling) in eusocial insects is one of the essential mechanism involved in the maintenance of the reproductive division of labor and thus it represents a major functional key of insect societies^17^. We observed for the first time that cuticular polar compounds of reproductives trigger body-shaking behavior in workers. Workers display more body-shaking in presence of live reproductives and extracts of reproductives than in presence of workers' extracts. In the termite *R. flavipes,* this behavior is a proximate of the presence of reproductives^18,43,44^ (also shown in this study). Thus, we bring behavioral evidence that reproductives recognition is mediated by cuticular polar compounds, with proteins and peptides good candidates as active compounds. Interestingly, the hydrocarbon heneicosane, a non-polar compound, has also been identified on the cuticle of male and female reproductives of *R. flavipes*, inducing body-shaking and antennation of workers^18^. However, some reproductives and some populations of *R. flavipes* do not produce heneicosane^64–66^. Therefore, more studies are needed to disentangle the potential complementary roles of heneicosane and polar compounds such as proteins on reproductives recognition. The presence of non-polar compounds is one of a few non-mutually exclusive explanations for the difference in effect between live reproductives and extracts we observed. Other chemical/physical cues, yet to be discovered could also be involved, which again could explain why live reproductives induce more body-shaking events than their cuticular polar extracts. For example, the cuticle structure itself could act as a carrier matrix with potentiation of the effect. Several other phenomena, alone or in concert, could account for the lower response elicited by a sand/cellulose paper support. First, proteins can adsorb on surfaces, and such adsorption on silica and cellulose material tends to be irreversible, which would decrease the apparent concentration, and thus the availability of the compounds for detection by workers. Second, some proteins may act synergistically^42^, so that an alteration of the ratio of some compounds might decrease their combined effect on workers. Taking all these considerations into account, future experiments aimed at identifying active proteins in behavioral assays should include the use of termite dummies, preferably foregoing the use of glass which bears the greatest adsorption risk. It should be noted that protein profiling was done on cuticular extracts directly, so that they more directly reflect the actual set of compounds present on the cuticle than extracts deposited on supports. Non-volatile compounds such as CPCs imply direct contact between the receptors and the substrate. When workers are exposed to a live reproductive or to an extract, in our setup it is not easy to determine if the workers exhibit body-shaking behavior immediately after contact, or if there is a delay in the behavior. The behavior could also be expressed after a short time-lapse and is not necessarily expressed directly during the contact event. Moreover, the receptor could be localized on the antennae or other body parts like the legs. It will be interesting to determine which receptors are involved in this behavior and what are their localization.

Time dependent comparisons showed an increase of body-shaking events at 24 h compared to 1 h in all treatments, except for reproductives' extracts where it was only marginally significant (Z = 8.5, *p* = 0.059; Fig. 3). This general increase, for both the mean values and also the data variances, may reflect a behavioral instability due to social isolation. Indeed, tested micro-nests are fragments of larger mother colonies displaying rich interactions. Therefore, workers might be more behaviorally disturbed after 24 h of isolation than after 1 h. At 24 h (Fig. 2B), the presence of reproductives still induce body-shaking but the reproductives extracts do not anymore. Alternatively, the short-lived effect of the extracts might be due to a lack of stability of the polar compounds over time. For proteins, this is a credible explanation as they would be susceptible to proteolysis by regurgitated gastric enzymes. On the other hand, some compounds may be long-lived: Turillazzi et al. reported long-lasting peptides on the cuticle and in the venom of *Polistes*: dominulin A (1854 Da) and dominulin B (1909 Da)^40^. It is possible that CPCs must be deposited repeatedly on substrates to maintain their effect, or that the cuticle is needed as a stabilizing carrier. Since extracts and focused workers were set up at once at the beginning of the experiments, we could not test the "stability over time of the extracts" separately from the "isolation time effect on workers", preventing conclusion on these hypotheses. It also would be interesting to determine the speed at which proteins/peptides in the extracts degrade or lose their structural integrities to the point of not be able to carry information anymore.

Cuticular polar extracts from all caste and sex are composed of a diverse mixture of proteins and peptides up to the 25 kDa range according to our mass spectrometry analyses. CPCs from reproductives are more diverse than those from workers' cuticle, with CPC-1330, CPC-4139 and CPC-4998 as main compounds separating both castes. In the paper wasp *P. dominulus*, foundress and their first emerged workers present clear specific CPCs (918 to 2679 Da)^37^. This caste specificity of CPCs was also observed in 3 termites species including our studied species *R. flavipes*^38^ (note that *R. santonensis* is a synonym of *R. flavipes*^67^). However, the compounds observed in female reproductives seems to be different from compounds in our study, except for the compound at *m/z* of 4141, which may correspond to CPC-4139 (*m/z* 4138.6). CPCs are also sex-specific for reproductives and workers in our study. Sex-specific CPCs are less common in the literature, but they have been described in a species phylogenetically close to termites, the cockroach *Leucophaea maderae*. In this species, males present two specific cuticular proteins (LMA-P18 and LMA-P22) progressively accumulated from ecdysis to sexual maturity^35^. The protein LMA-P22 is supposed to be involved in sexual behavior and belong to the lipocalin family known to bind hydrophobic compounds like pheromones^68^. In our study, among the 87 major CPCs we identified in reproductives 19 CPCs specific to females and 18 to males, including 3 CPCs more abundant in females than males, suggesting a function of CPCs in sex signaling and caste regulation. Indeed, the production of reproductives in *R. flavipes* is under a dual sex-specific regulation^22^. While the presence of female reproductives inhibits the production of new female reproductives, it also stimulates the molting of male workers into new male reproductives. A reciprocal phenomenon is observed for male reproductives. In workers, only females present sex-specific CPCs with 2 compounds (CPC-4139 and CPC-4158). Interestingly, CPC-4139 is the most abundant CPC in reproductives, whereas in workers, it is found in very low abundance, in only 2 samples out of 30. This low abundance and frequency may reflect the presence of female workers starting to develop into reproductives. Removing those individuals from the dataset did not change the results on group separations and on the identification of caste/sex specific CPCs. We also noticed that a few reproductives (particularly males) present CPC profiles close to workers (Fig. 5), suggesting that these males may be new immature reproductives. It would be interesting to monitor the maturation of the CPC profiles for individuals engaged into reproductive differentiation, such as workers and nymphs, during the different molting phases, as well as to track body-shaking behaviors according to the sex of the reproductives. Overall, these results are consistent with the possible role of CPCs in reproductive recognition, sex signaling and caste regulation.

In insects, secreted and cuticular proteins/peptides have been mainly identified to possess antimicrobial activities^37,62,69^. Mass spectrometry proteomics explorations allowed us to confirm that polar extracts contain proteins and may bear post-translational modifications such as glycosylations. Our preliminary fragmentation study shows that CPC-10056 is an ortholog of the midgut enzyme protein G12, also identified in several termite species. Interestingly, at least one digestive enzyme is known to be involved in the reproductive division of labor in the termite *C. secundus*^70^: the *Neofem2* gene encodes a wood digestion enzyme (β-glucosidase). Indeed, silencing *Neofem2* in queens induce fights between workers for the royal succession in queenless colonies. Moreover, this β-glucosidase is involved in egg recognition in the termite *R. speratus*^63^, and in the mating behavior of the cockroach *Leucophaea maderae*^71^. Altogether, these observations suggest that the function of the β-glucosidase may have evolved from wood digestion to pheromonal caste regulation in cockroaches and termites, even though other chemicals could contribute to these pheromonal functions^18,63,70,71^.

Future studies will be needed to sequence and identify reproductive-specific CPCs and to test their behavioral functions, either individually or in synergy, to determine if they play a role in the regulation of social interactions in insects, as observed in vertebrates or aquatic organisms^3^. Sex-specific variations observed in reproductives also open new fields of inquiry for the potential role of proteins in the dual sex-specific regulation of the reproductives which is occurring in subterranean termites^22,72^. The scope of our study is limited by the fact that sand-deposited polar extracts may be depleted in some proteins and other polar molecules that may act as synergistic compounds. Despite these limitations, the combination of protein profiling with behavioral bioassays represents a powerful tool to explore unknown aspects of insect communications, which when further correlated through the identification of the active proteins, could help to answer unresolved questions about social insect communication.

## Supplementary Information

Supplementary Figures.

## Data Availability

The datasets generated during the current study are available in the Zenodo repository (https://zenodo.org/record/7319584).
