# Supplementary material for: Reproductives signature revealed by protein profiling and behavioral bioassays in termite
Source: Sci Rep. 2023 May 1;13:7070. doi: 10.1038/s41598-023-33252-6 (PMC10151321; doi:10.1038/s41598-023-33252-6)

Supplementary figures

Sup. Fig. 1: Compounds repartition across castes and sexes of the first-quatile of the 87 major CPCs. CPCs were attributed to a group if more than one individual present the compound. For the full list of CPCs across castes and sexes see table 1.

Supplementary Figure 1

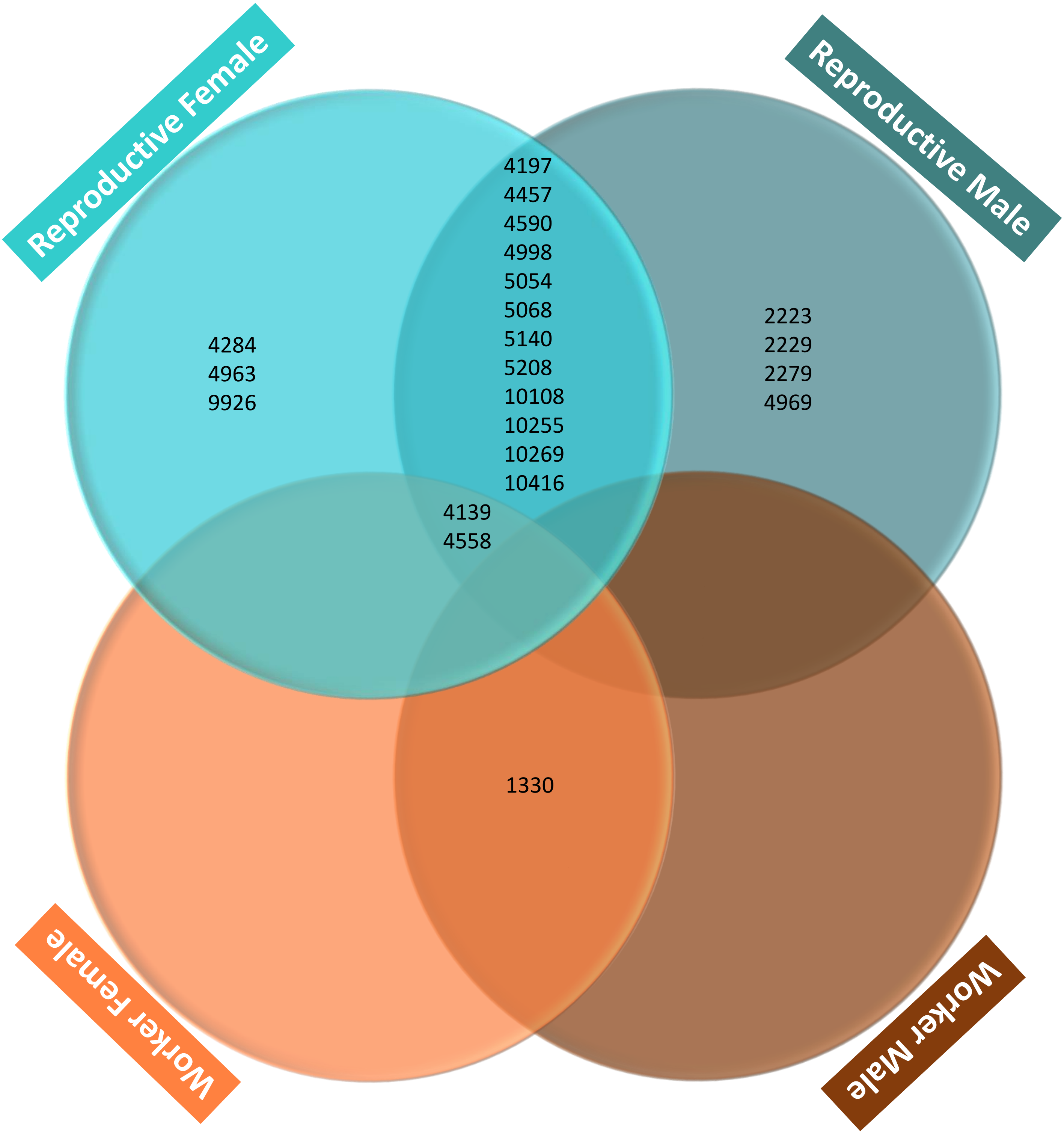

Sup. Fig. 2: Averaged mass spectra of cuticular polar extracts from MALDI-TOF analyses. Spectra are shown in positive/negative display for reproductives versus workers with spectra summed for both sexes. The spectra are based on a sum of 30 spectra for each caste. Annotated peaks represented in bold are the first-quartile of the 87 major CPCs identified as discriminant by the PLS-DA analyses with VIP values greater than 1. Note that the 3 major CPCs 4428, 10056 and 10196 are also represented in the figure to support the discussion even if they are not in the first-quartile (see the result section for details). See table 1 for the entire list of compounds.

Supplementary Figure 2

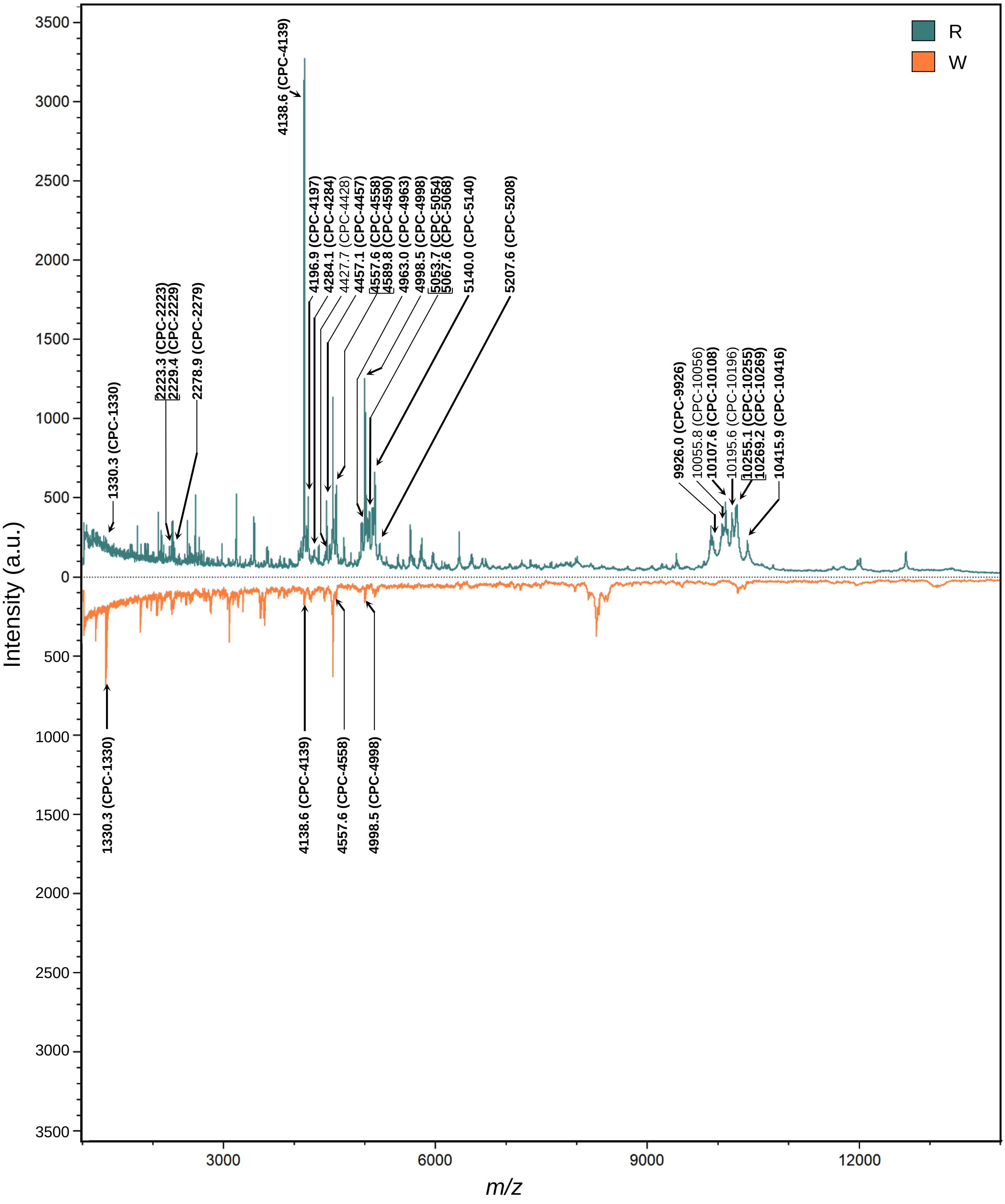

Supplement: Supplementary file 1 — Supplementary Figures. [file 41598_2023_33252_MOESM1_ESM.pdf]
